# Co_2_P_2_O_7_ Microplate/Bacterial Cellulose–Derived Carbon Nanofiber Composites with Enhanced Electrochemical Performance

**DOI:** 10.3390/nano11082015

**Published:** 2021-08-06

**Authors:** Likkhasit Wannasen, Wiyada Mongkolthanaruk, Ekaphan Swatsitang, Prasert Pavasant, Supree Pinitsoontorn

**Affiliations:** 1 Institute of Nanomaterials Research and Innovation for Energy (IN–RIE), Department of Physics, Faculty of Science, Khon Kaen University, Khon Kaen 40002, Thailand; likkhasit123@hotmail.co.th (L.W.); ekaphan@kku.ac.th (E.S.); 2 Department of Microbiology, Faculty of Science, Khon Kaen University, Khon Kaen 40002, Thailand; wiymon@kku.ac.th; 3 Thai Roong Ruang Sugar Group, Bangkok 10120, Thailand; supersert@gmail.com

**Keywords:** Co_2_P_2_O_7_, bacterial cellulose, carbon nanofiber, electrochemical properties, supercapacitor

## Abstract

Nanocrystalline Co_2_P_2_O_7_ and carbon nanofiber (Co_2_P_2_O_7_/CNFs) composites with enhanced electrochemical performance were obtained by calcination after a hydrothermal process with NH_4_CoPO_4_∙H_2_O/bacterial cellulose precursors under an argon atmosphere. SEM images showed that the CNFs were highly dispersed on the surfaces of Co_2_P_2_O_7_ microplates. The diagonal size of the Co_2_P_2_O_7_ plates ranged from 5 to 25 µm with thicknesses on a nanometer scale. Notably, with the optimal calcining temperature, the Co_2_P_2_O_7_/CNFs@600 material has higher specific micropore and mesopore surface areas than other samples, and a maximal specific capacitance of 209.9 F g^−1^, at a current density of 0.5 A g^−1^. Interestingly, CNF composite electrodes can enhance electrochemical properties, and contribute to better electrical conductivity and electron transfer. EIS measurements showed that the charge–transfer resistance (R_ct_) of the CNF composite electrodes decreased with increasing calcination temperature. Furthermore, the Co_2_P_2_O_7_/CNF electrodes exhibited higher energy and power densities than Co_2_P_2_O_7_ electrodes.

## 1. Introduction

With the rapidly increasing global warming problem around the world, air pollutants that aggravate this problem, such as carbon monoxide, nitrogen oxides, and carbon dioxide, are produced by burning gasoline [1,2]. Nowadays, batteries for hybrid electric vehicles and electric vehicles have been increasingly in demand for their high performance energy storage, long life cycles, safety, and environmental friendliness [3]. Hybrid Li–ion batteries and supercapacitors have an important role in electric vehicles due to their high energy and power densities, respectively [4,5]. Supercapacitors are especially attractive. Their use has increased in various applications because of their high power density, fast charge–discharge time and long life cycles compared to other energy storage devices [6]. It is well established that supercapacitors store energy through two mechanisms: ion adsorption (electric double layer capacitors, EDLCs) and fast surface redox reactions (pseudo–capacitance) [6,7]. The key performance parameters of capacitance in supercapacitors includes their available surface area, pore structure, electrical conductivity, redox reactions, and the use of an aqueous electrolyte [8]. Commonly, the electrode material of a supercapacitor consists of activated carbon, which can be converted into many forms such as buckyballs, nanotubes, nanobuds, and nanofibers. This is because of its large specific surface area, high porosity, high conductivity, high thermal stability and low cost [9,10,11,12]. For example, graphene oxide loaded in a poly vinylidene fluoride–co–hexafluoropropylene by the innovative preparation methods showed the high energy density for an aerogel supercapacitor [13]. Recently, various metal oxides such as RuO_2_ H_2_O [14], Mn_3_O_4_ [15] MnO_2_ [16], Fe_3_O_4_ [17], Ni(OH)_2_ [18], and Co_3_O_4_ [19], which can exhibit a Faradaic charge transfer between electrode and electrolyte, have been widely investigated for use as electrodes of pseudo–capacitors.

Metal phosphate–based materials for high electrochemical performance have become increasingly rigid in supercapacitors. These materials use their porous structure for adsorption–desorption of ions with redox reactions between an electrode and electrolyte [20,21,22,23]. Recently, cobalt pyrophosphates (Co_2_P_2_O_7_) with various morphologies have been extensively investigated as advanced electrode materials in electrochemical energy storage. They exhibit deep intercalation in a lattice, fast/reversible surface redox reactions and a layering of metal phosphonates and phosphate [24,25,26]. Additionally, Co_2_P_2_O_7_ has a high level of mesopores and a favorable Faradic process as electrodes in pseudo–capacitors [24]. It is established that the performance of supercapacitors is relative to their morphology, size, surface chemistry, porosity, and type of electrolyte employed. These properties depend on the preparation methods and conditions used [23]. However, there is a need to increase the energy storage capacity limits. Carbon–based composites play an important role in enhancing the electrochemical properties of these materials due their electron and ion transfer networks, which serve to increase electrolyte contact and conductivity [27,28,29,30]. Various materials have been obtained by calcination after synthesis of a NH_4_CoPO_4_∙H_2_O micro/nanostructure with different morphologies [24,26]. Nevertheless, the carbon–supported electrochemical properties of Co_2_P_2_O_7_ have not been reported.

Generally, carbon nanofibers (CNFs) are synthesized using a variety of biomass types and bio–wastes. They are made by pyrolysis of natural cellulose–based materials (cellulose biomass and bacterial cellulose) [31,32,33]. CNFs from a variety of biomass components (e.g., cellulose and lignin) are actively being studied as renewable energy storage materials due to their great abundance, low cost, biocompatibility, and eco–friendly nature [33,34,35]. However, CNFs from biomass are micro–sized and may contain impurities in the form of lignin, hemicellulose, and other biopolymers [36]. On the other hand, bacterial cellulose (BC), synthesized from various types of bacteria [37,38], has been shown to exhibit interesting properties that suggest it can replace cellulose from other biomass types. For example, BC has a 3D porous structure consisting of a polysaccharide–based nanofiber network composed of linear chains with a molecular formula of (C_6_H_10_O_5_)_n_. Remarkably, combining CNF derived from BC and metal oxides plays an important role in the electrical conductivity of these materials. It can correct and compensate for the poor electrical conductivity and low current density of charge processes that are the disadvantages of metal oxides [30,39].

In this paper, Co_2_P_2_O_7_/CNF composites were obtained by calcination after a hydrothermal process using a NH_4_CoPO_4_∙H_2_O/BC substrate under an argon atmosphere at 400, 600, and 800 °C for 2 h. The present study aims to improve electrochemical efficiency with carbon nano–fiber composites for conductivity enhancement, develop an electron transfer network, and improve electrolyte contact. The phases and components of conductive CNFs were controlled by adjusting calcination temperature. Moreover, increased calcination temperature during synthesis can improve the conductivity of samples by enhancing electron transfer between the electrodes and electrolyte. As a result, with an optimized calcination temperature, Co_2_P_2_O_7_/CNFs@600 exhibits a large hysteresis loop, indicating higher specific micro– and mesoporous surface areas than other samples. Additionally, Co_2_P_2_O_7_/CNFs@600 also showed a maximum specific capacitance of 209.9 F g^−1^ at a current density of 0.5 A g^−1^.

## 2. Materials and Methods

### 2.1. Biosynthesis of Bacterial Cellulose (BC)

*Komakataeibacternataicola* was used to prepare BC in a culture medium. The culture medium consisted of 10 g of D–glucose and 1 g of yeast extract dissolved in 1 L of de–ionized (DI) water. After 14 days of incubation at 30 °C, the obtained BC hydrogels were boiled at 100 °C in DI water. Then, the BC hydrogels were immersed in 0.5 M and 5 wt% of NaOH for 15 min and 24 h, respectively. Subsequently, DI water was used to wash BC hydrogels several times until pH 7 was achieved. Subsequently, the BC hydrogels were pulped using a mechanical homogenizer (EM–ICEPOWER–600W–SHARP, Western appliances Co. Ltd., Bangkok, Thailand) operated at ~10,000 rpm until complete homogenization was accomplished. The resulting BC homogenate was filtered through Whatman #1 filter paper, passing particles with diameters of less than ~11 μm, which were then used to produce BC products.

### 2.2. Preparation of Co_2_P_2_O_7_/CNFs Composites

**Figure 1:**
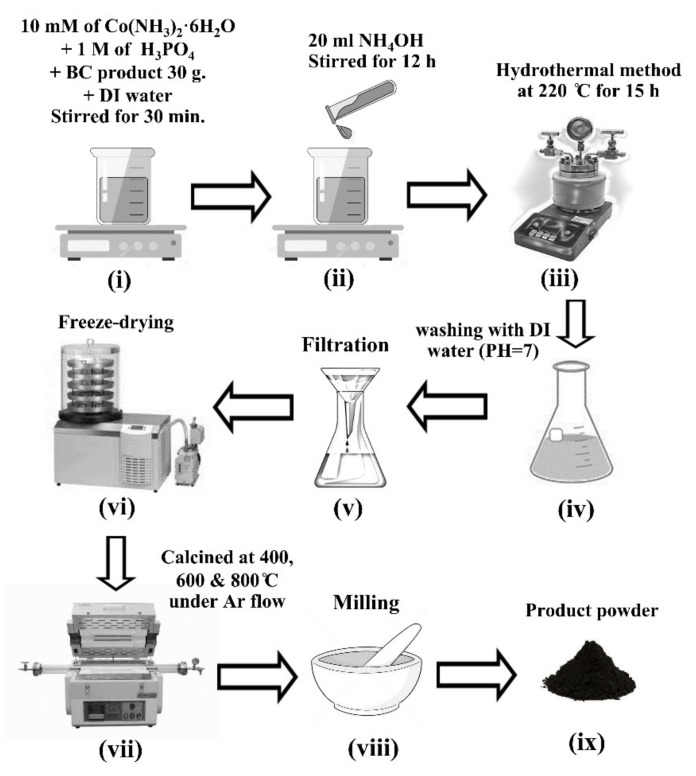
A schematic illustration for the preparation of Co_2_P_2_O_7_/CNFs composite fabrication (**i**) Stoichiometric amounts of raw materials, (**ii**) Adding NH_4_OH, (**iii**) Hydrothermal process, (**iv**) Washing, (**v**) Filtration, (**vi**) Freeze–drying, (**vii**) Calcination, (**viii**) Milling and (**ix**) Product powder.

For synthesis of NH_4_CoPO_4_∙H_2_O/BC composites, 0.01 M of cobalt (II) nitrate hexahydrate (Co(NO_3_)_2_∙6H_2_O, M_w_ = 291.03, Aldrich, ≥97%) and 1 M phosphoric acid (H_3_PO_4_, M_w_ = 97.99, RCI) were dissolved in 130 mL of DI water under strong mechanical stirring for 30 min. Then, 30 g of BC product was added, followed by continuous stirring for 3 h (Figure 1(i)). Then, 20 mL of aqueous ammonia (NH_3_, Mw = 17.03, RCI) was added dropwise and continuously stirred for a further 12 h (Figure 1(ii)). Subsequently, the solution was transferred to a 200 mL Teflon stainless steel autoclave and subjected to a hydrothermal treatment at 220 °C for 15 h (Figure 1(iii)). After this step, the NH_4_CoPO_4_∙H_2_O/BC products were washed several times with deionized water until they reached a pH of 7 and subsequently filtered, as described in Section 2.1, to obtain NH_4_CoPO_4_∙H_2_O/BC hydrogels (Figure 1(iv,v)). Then, the NH_4_CoPO_4_∙H_2_O/BC aerogels were obtained by freeze–drying in a bulk tray dryer (GAMMA 2–16 LSC, CHRIST, Osterode am Harz, Germany) (Figure 1(vi)).

**Figure 2:**
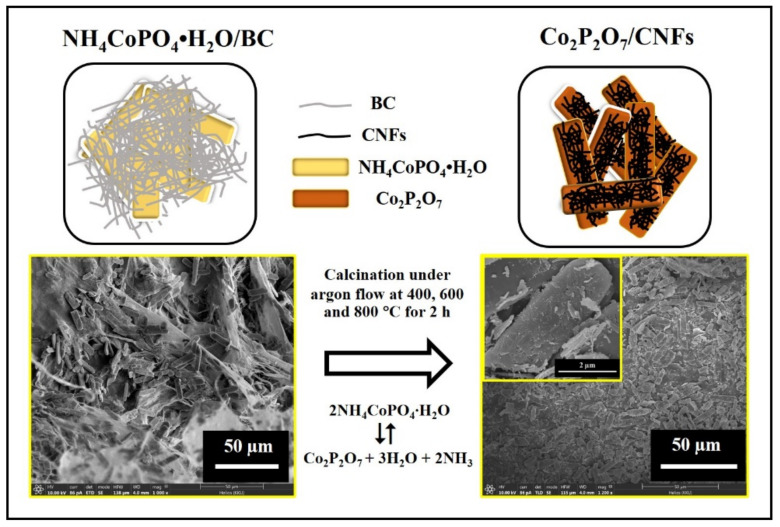
Schematic illustration with FE–SEM images for conversion of NH_4_CoPO_4_∙H_2_O/BC aerogels to Co_2_P_2_O_7_/CNFs.

To convert NH_4_CoPO_4_∙H_2_O/BC aerogels to Co_2_P_2_O_7_/CNFs, they were calcined in a quartz–tube furnace under an argon flow at 400, 600, and 800 °C for 2 h with a heating rate of 5 °C min^−1^ (Figure 1(vii,viii)). Subsequently, the samples were ground in a mortar and pestle. The Co_2_P_2_O_7_/CNF products are referenced according to their calcination temperature as Co_2_P_2_O_7_/CNFs@400, Co_2_P_2_O_7_/CNFs@600, and Co_2_P_2_O_7_/CNFs@800 for NH_4_CoPO_4_∙H_2_O/BC aerogels calcined at 400, 600, and 800 °C, respectively. A schematic illustration with FE–SEM images for the conversion of NH_4_CoPO_4_∙H_2_O/BC aerogels to Co_2_P_2_O_7_/CNFs is shown in Figure 2.

### 2.3. Characterizations

Thermal gravimetric analysis (TGA) and derivative thermogravimetry (DTG) (STA7200, HITACHI, Tokyo, Japan) were used to determine the phase transformations and CNF components. The samples (~5–10 mg) were loaded into platinum pans and heated to 850 °C at a heating rate 10 °C∙min^−1^ under a N_2_ atmosphere (flow rate 150 mL min^−1^). Structural identification of crystalline material was carried out via X–ray diffraction (XRD) using Cu K_α_ radiation employing PANalytical equipment (Empyrean, Almelo, The Netherland). The morphology and dispersive elemental components of samples were characterized using field emission scanning electron microscopy (FE–SEM, Helios Nanolab G3 CX, FEI, Brno, Czech Republic) and EDS–mapping (X–MaxN–80, Oxford), respectively. Additionally, transmission electron microscopy (TEM, TECNAI G^2^ 20, FEI, Brno, Czech Republic) with bright field images, selected area electron diffraction (SAED) patterns and energy–dispersive X–ray spectroscopy (EDXS) were used to identify the phase, morphology, and elemental content, confirming the results obtained from XRD and FE–SEM. Moreover, the specific surface area (SSA) was determined through a Brunauer–Emmett–Teller (BET) method. Pore size distributions (PSD) were obtained using a Barrett–Joyner–Halenda (BJH) method that employed N_2_ adsorption–desorption (miniX, BELSORP, Osaka, Japan). All samples were degassed at 120 °C under a vacuum for 12 h before measurements were made. Fourier transform infrared spectrophotometry (FT–IR) (TENSOR27, Bruker, Osaka, Japan) was used to identify the functional groups of the materials.

### 2.4. Electrochemical Measurements

The electrochemical properties of the sample were determined in a 3M KOH electrolyte using cyclic voltammetry (CV), galvanostatic charge–discharge (GCD), and electrochemical impedance spectra (EIS). A three–electrode configuration was employed with a platinum counter electrode and a Ag/AgCl reference electrode. Working electrodes were prepared from Co_2_P_2_O_7_/CNFs using a mixture of the active material (90 wt%) combined with polyvinylidene fluoride (10 wt%) to fabricate an *N*–Methyl–2–pyrrolidone (NMP) solution. Then, this mixed solution was coated on a 1 × 1.5 cm^2^ Ni foam substrate over a 1 × 1 cm^2^ coated area followed by drying in an oven at 80 °C for 24 h. Finally, the coated Ni foam specimens were subjected to a 5 MPa pressure under uniaxial compression.

A CORRTEST (CS350 Potentiostat /Galvanostat, Corrtest instruments, Wuhan, China) electrochemical workstation was used for all electrochemical experiments. The CV measurement was performed at room temperature within the voltage a range of −0.2 to +0.4 V at scan rates ranging from 5–200 mVs^−1^. The GCD method is an electrochemical analysis to determine specific capacitance and cyclic stability of an electrode. The specific capacitance (C_s_) was calculated using Equations (1) and (2) for CV and GCD, respectively. The electrical conductivity of the electrodes was investigated over the frequency range of 0.01 Hz–100 kHz with an EIS method.

The specific capacitance (C_s_) from the CV method, in terms of Farads/gram, can be calculated following Equation (1).

(1) $$C_{s}\left(\frac{F}{g}\right)=\frac{\int_{E2}^{E1}\mathrm{IdV}}{\mathrm{mv}\Delta E}$$

where the ∫IdV term refers to the area under a CV curve (cathodic), m refers to the weight of the active material within an electrode, v is the scan rate and ΔE refers to the voltage window (E_2_–E_1_).

The specific capacitance (C_s_) of GCD method was calculated using Equation (2).

(2) $$C_{s}\;\left(\frac{F}{g}\right)=\frac{i\;\times \Delta t}{\Delta V\times m}$$

where ***i*** (A) is the applied current, Δt (s) is the discharge time, ΔV (V) is the operating potential window of charge or discharge in Volts and m (g) is the mass of active materials.

The energy (E) and power densities (P) of the electrodes were calculated following Equations (3) and (4), respectively [40].

(3) $$E\left(\frac{\mathrm{Wh}}{\mathrm{kg}}\right)=\frac{C_{\mathrm{sp}}{\left(\Delta V\right)}^{2\;}}{7.2}$$

(4) $$P\left(\frac{W}{\mathrm{kg}}\right)=\frac{E\times 3600}{\Delta t}$$

where ΔV (V) is the operating potential window of charge or discharge in Volts, i (A) is the applied current, Δt (s) is the discharge time, and m (g) is the mass of active materials.

## 3. Results and Discussion

### 3.1. TGA–DTG

**Figure 3:**
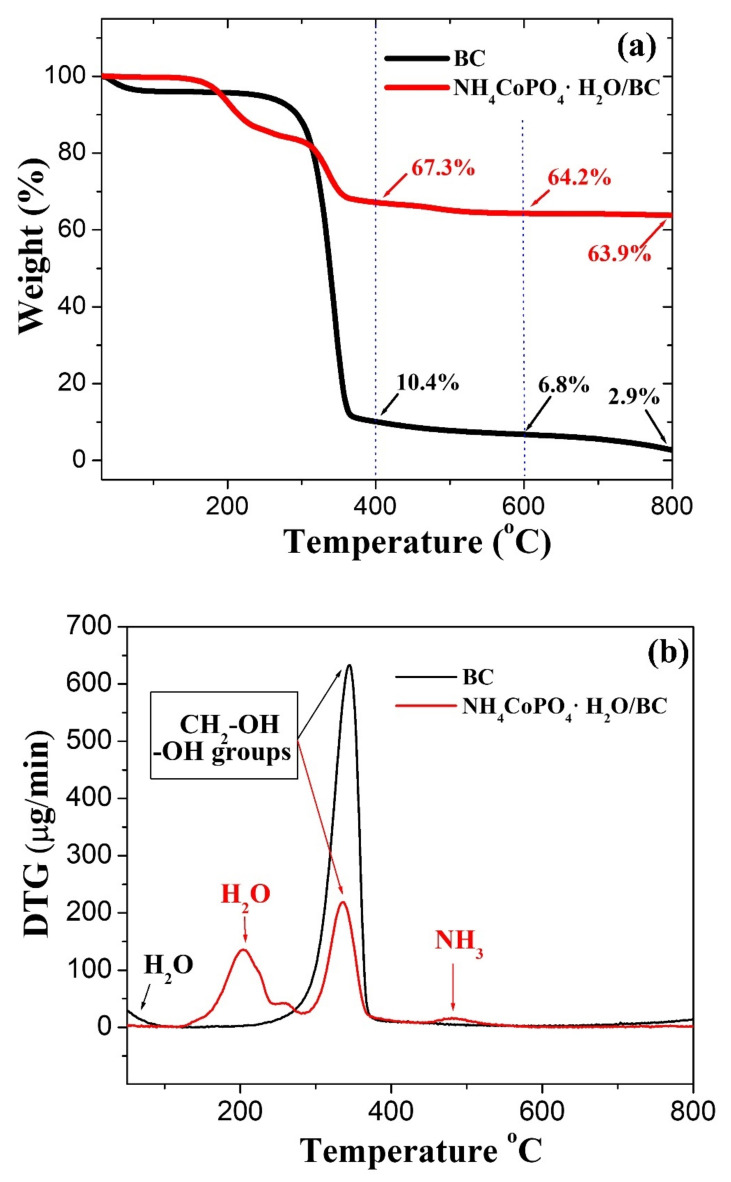
(**a**) TGA and (**b**) DTG curves of NH_4_CoPO_4_∙H_2_O/BC and BC.

The phase transition of NH_4_CoPO_4_∙H_2_O/BC to Co_2_P_2_O_7_/CNFs was determined using TGA/DTG measurements. The TGA and DTG curves of NH_4_CoPO_4_∙H_2_O/BC and BC from 50 to 850 °C can be seen in Figure 3. From the TGA/DTG curves of NH_4_CoPO_4_∙H_2_O/BC, three significant weight loss temperature ranges were observed, 150–300 °C, 300–350 °C and 350–550 °C, corresponding to three main peaks of the DTG curve. First, an initial weight loss of about 16% at room temperature to 300 °C resulted from moisture loss or removal of water from the lattice following Equation (5). It should be noted that the DTG peak for the evaporation of water molecule occurs at different positions between BC and NH_4_CoPO_4_∙H_2_O/BC samples. The water can be easily evaporated from the BC structure, so that the DTG peak around 100 °C is observed [37,38]. On the other hand, NH_4_CoPO_4_∙H_2_O is a layered structure in which water molecules are intercalated [24,26]. Therefore, it is more difficult to remove water molecules, and the DTA peak around 200 °C is observed for the NH_4_CoPo_4_H_2_O/BC sample.

Second, a major weight loss of about 17.3% was ascribed to decarboxylation and decarbonylation reactions in BC and the hydrogen phosphate salts in NH_4_CoPO_4_∙H_2_O. Last, a small weight loss of about 3% was explained by the loss of an ammonium complex leading to the formation of gaseous ammonia, which can be described by Equation (6). Furthermore, no signal weight loss or thermal effect were found in the TGA–DTG curves above 550 °C [24]. Clearly, cobalt pyrophosphate composite carbon nanofibers (Co_2_P_2_O_7_/CNFs) can be obtained by calcination of NH_4_CoHPO_4_/BC under an argon atmosphere at 400, 600 and 800 °C. The conversion of NH_4_CoHPO_4_/BC to Co_2_P_2_O_7_/CNFs follows Equation (7).

NH_4_CoPO_4_∙H_2_O → NH_4_CoPO_4_ +H_2_O (5)

NH_4_CoHPO_4_ → CoHPO_4_ + NH_3_ (6)

2NH_4_CoPO_4_∙H_2_O → Co_2_P_2_O_7_ + 3H_2_O + 2NH_3_ (7)

From TGA data, the ratio of the CNF content in Co_2_P_2_O_7_/CNFs was calculated using the residual content of CNFs at 400, 600, and 800 °C. The CNF components in the Co_2_P_2_O_7_/CNFs were 15.45%, 10.59%, and 4.54% for Co_2_P_2_O_7_/CNFs@400, Co_2_P_2_O_7_/CNFs@600 and Co_2_P_2_O_7_/CNFs@800, respectively. These methods confirm that the weight (%) of CNFs in Co_2_P_2_O_7_/CNFs decreased with increasing calcination temperature.

### 3.2. XRD

**Figure 4:**
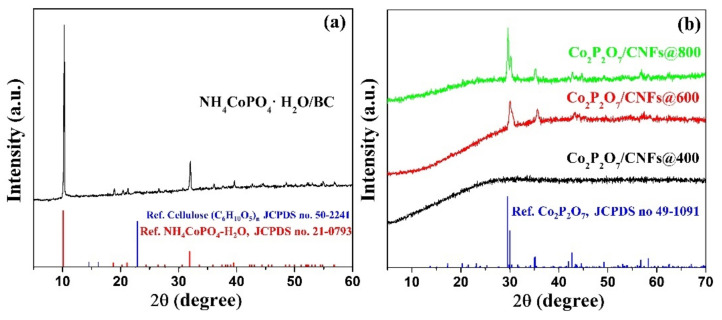
XRD results of (**a**) NH_4_CoPO_4_–H_2_O/BC compared to the references for NH_4_CoPO_4_∙H_2_O, cellulose and (**b**) Co_2_P_2_O_7_/CNFs@400, Co_2_P_2_O_7_/CNFs@600, and Co_2_P_2_O_7_/CNFs@800 compared to the references for Co_2_P_2_O_7_.

The XRD results of NH_4_CoPO_4_∙H_2_O/BC composites are shown in Figure 4a. All the diffraction peaks of NH_4_CoPO_4_∙H_2_O/BC are indexed well to the orthorhombic phase in NH_4_CoPO_4_∙H_2_O, within space group *Pmn*2_1,_ referring to standard data (JCPDS No. 21–0739) [41]. The high intensity peak at a 2θ value of 10.1° can be indexed to the (010) plane, indicating that the sample has a highly crystalline structure. Moreover, the broad peaks at 2θ = 23.0° correspond to the (200) diffraction peaks of BC (JCPDS No. 50–2241) [42], suggesting that the BC fiber was mixed in NH_4_CoPO_4_∙H_2_O and completely converted into CNFs by the calcination method. For the Co_2_P_2_O_7_ and CNF composite, the XRD pattern of Co_2_P_2_O_7_/CNFs are shown in Figure 4b. The XRD pattern of the Co_2_P_2_O_7_/CNFs@400 sample exhibits an amorphous phase with no detectable diffraction peaks. It can be clearly seen that the Co_2_P_2_O_7_/CNFs@600 and Co_2_P_2_O_7_/CNFs@800 samples indicate a monoclinic structure. This result is consistent with the standard data of Co_2_P_2_O_7_ (JCPDS No. 49–1091) [24]. As a result, the peak intensities of these samples increase with calcination temperature. It is notable that the crystallinity of calcined Co_2_P_2_O_7_/CNFs@800 is higher than that of Co_2_P_2_O_7_/CNFs@600. Moreover, three dominant peaks at 14.3°, 16.6°, and 22.5° reveal a Type–I cellulose structure of BC that can be indexed to the $\left(1\overset{-}{1}0\right)$, (110) and (200) planes, respectively [42]. Additionally, the broad diffraction peaks of CNFs@400, CNFs@600, and CNFs@800 positioned at 5–40° can be indexed to amorphous CNFs for all calcination temperatures, as shown in Figure S1 (Supplementary Information).

### 3.3. FE–SEM

**Figure 5:**
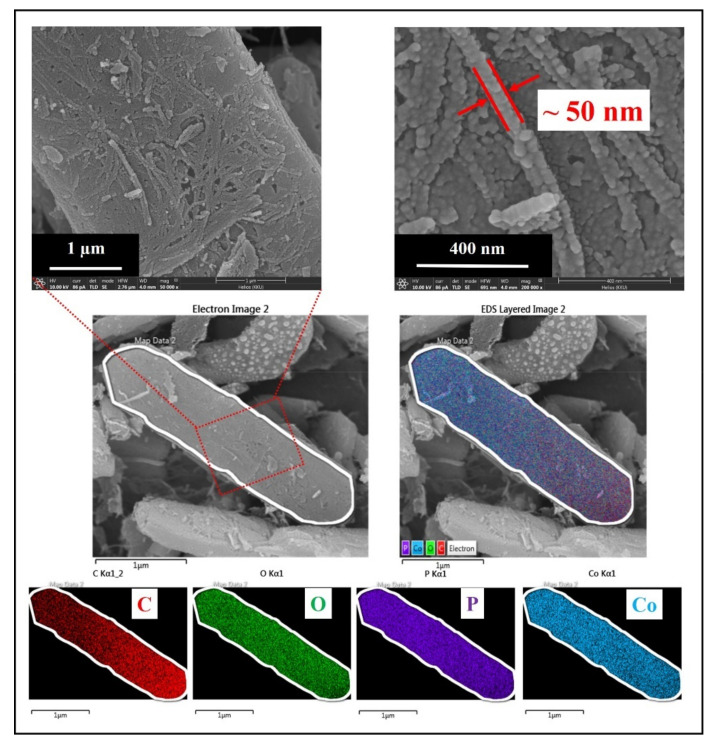
FE–SEM images and selected area elemental mappings of Co_2_P_2_O_7_/CNFs@600.

Figure 5 presents FE–SEM images and elemental mappings of Co_2_P_2_O_7_/CNFs@600. The FE–SEM images show that the morphology of Co_2_P_2_O_7_ is plate–shaped and the diagonal sizes of the Co_2_P_2_O_7_ particles range from 2 to 25 nm with thicknesses on a nanometer scale. Moreover, CNFs from BC are scattered on the surfaces of the Co_2_P_2_O_7_ plates. It can be clearly seen that the diameters of the CNFs are about 50 nm. The dispersion of CNFs and elemental distribution of components of the samples were investigated using an energy dispersive mode (EDX) in FE–SEM. Image J software was employed to determine the diagonal size distribution of Co_2_P_2_O_7_/CNF samples, as shown in Figure S2 (Supplementary Information). From these results, the average diagonal sizes were 9.28, 11.85, and 12.90 µm for Co_2_P_2_O_7_/CNFs@400, Co_2_P_2_O_7_/CNFs@600, and Co_2_P_2_O_7_/CNFs@800, respectively. Moreover, the average diagonal size slightly increased with calcination temperature [43]. Furthermore, the selected area elemental mappings are shown at the bottom of Figure 5. It can be clearly seen that the carbon (C) atoms of CNFs were homogeneously dispersed on the surfaces of Co_2_P_2_O_7_. Clearly, oxygen (O), phosphorus (P), and cobalt (Co) atoms were homogeneously scattered throughout the Co_2_P_2_O_7_ microplates.

### 3.4. TEM

**Figure 6:**
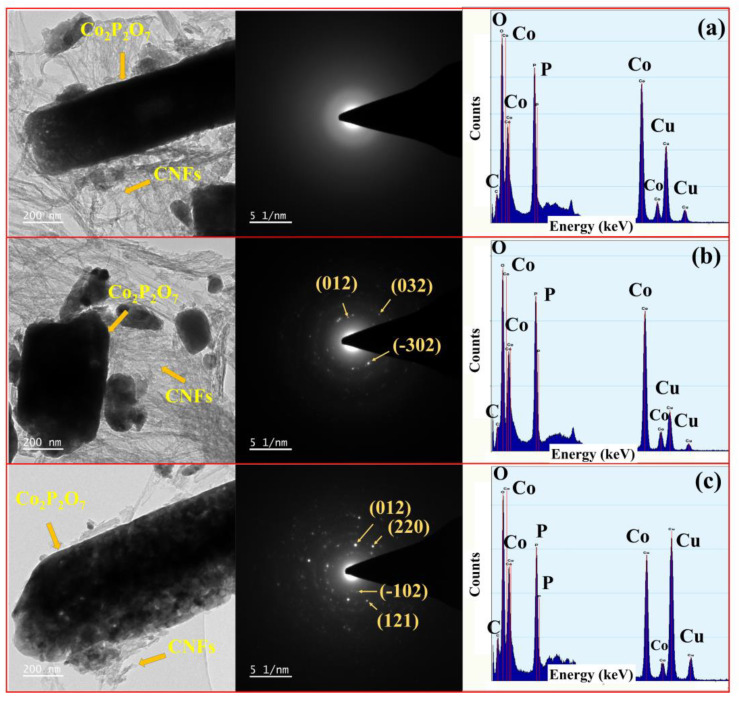
TEM bright field image, selected area electron diffraction (SAED) pattern and EDXs spectra of (**a**) Co_2_P_2_O_7_/CNFs@400, (**b**) Co_2_P_2_O_7_/CNFs@600 and (**c**) Co_2_P_2_O_7_/CNFs@800.

Figure 6 presents typical bright field TEM images, SAED patterns, and the EDX characterizations of (a) Co_2_P_2_O_7_/CNFs@400, (b) Co_2_P_2_O_7_/CNFs@600, and (c) Co_2_P_2_O_7_/CNFs@800. TEM bright field imagery of all samples revealed that CNFs clustered were around Co_2_P_2_O_7_ with crystallite sizes on a nanometer scale, suggesting a significant function of CNFs as an electrical conductivity promoter. Additionally, the typical SAED patterns of Co_2_P_2_O_7_/CNFs@600 and Co_2_P_2_O_7_/CNFs@800 (Figure 6b,c) show randomly scattered spotty points and halos due to a combination of a small amount of a polycrystalline portion with a larger portion of crystallite phase in all samples. As the calcination temperature was increased to 800 °C, the intensity of spotty points became greater than the sample calcined at 600 °C, indicating larger particles and high crystallinity in the 800 °C sample. Moreover, the SAED pattern of Co_2_P_2_O_7_/CNFs@400 in Figure 6a shows a diffuse amorphous diffraction ring. These SAED results are in good agreement with XRD findings showing an amorphous phase in Co_2_P_2_O_7_/CNFs@400 with a monoclinic structure in Co_2_P_2_O_7_/CNFs@600 and Co_2_P_2_O_7_/CNFs@800. Further detailed EDXs elemental analysis and the EDXs peaks reveal that the Co_2_P_2_O_7_/CNFs are composed of Co, C, P, and O with Cu peaks from a copper grid used in the analysis. These results illustrate the atomic weight (%) of carbon decreased with increasing calcination temperature, in good agreement with TGA results. For example, the molar ratio of Co:P:O (Co_2_P_2_O_7_@600) is approximately 13.5:15.9:53.8 (or 1:1.2:4.0), which is slightly different from the theoretical elemental ratio of 1:1:3.5 determined from the chemical formula. The atomic weights (%) of all elements were measured and the obtained results are summarized in Table 1.

### 3.5. FT–IR

**Figure 7:**
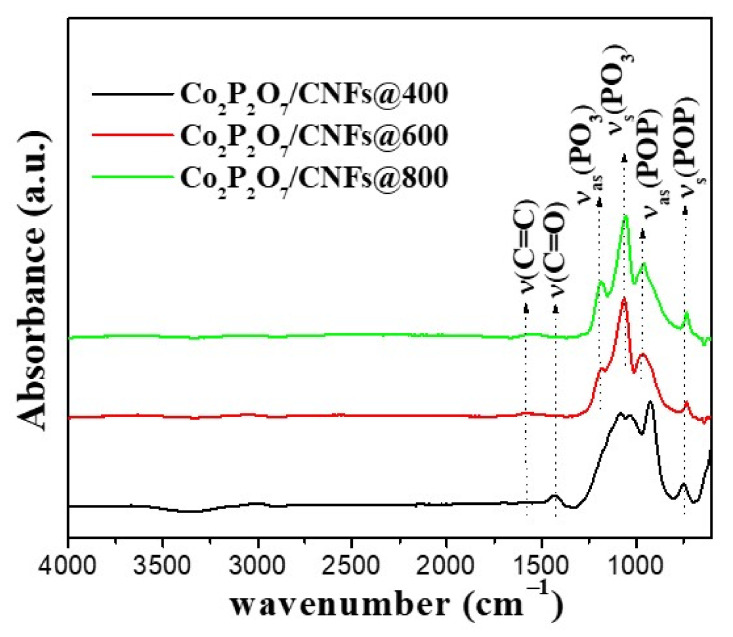
FT–IR spectra of Co_2_P_2_O_7_/CNFs@400, Co_2_P_2_O_7_/CNFs@600 and Co_2_P_2_O_7_/CNFs@800.

FT–IR spectroscopy was used to characterize the chemical bonding of the Co_2_P_2_O_7_/CNF samples. From the FT–IR spectra in Figure 7 for Co_2_P_2_O_7_/CNFs@600 and Co_2_P_2_O_7_/CNFs@800, it was observed that the peaks of samples at 740, 960, 1050, and 1200 cm^−1^ can be attributed to symmetric stretch (ν_s_, POP) bridge vibrations, asymmetric stretch (ν_as_, POP) bridge vibrations, symmetric vibrations (ν_s_, PO_3_) and asymmetric vibrations (ν_as_, PO_3_), respectively [44]. From these results, it was revealed that a [P_2_O_7_]^4–^ group in the calcined samples at 600 and 800 °C indicates that a pyrophosphate structure was formed. For samples calcined at 400 °C, the broadened bands of Co_2_P_2_O_7_/CNFs@400 appear in these FT–IR spectra, indicating formation of an amorphous phase with no peak of P–O–P or PO_3_ bridge stretching vibrations in Co_2_P_2_O_7_/CNFs@400. [18,45]. For peaks of CNF composites, C=O (1400 cm^−1^) and C=C (1620 cm^−1^) stretching vibrations were confirmed for carbon bonding in the structure of the CNFs [46,47]. These FT–IR results were confirmed and support the XRD, SEM, and TEM results, indicating Co_2_P_2_O_7_ composite CNFs.

### 3.6. Nitrogen Adsorption–Desorption Measurements

**Figure 8:**
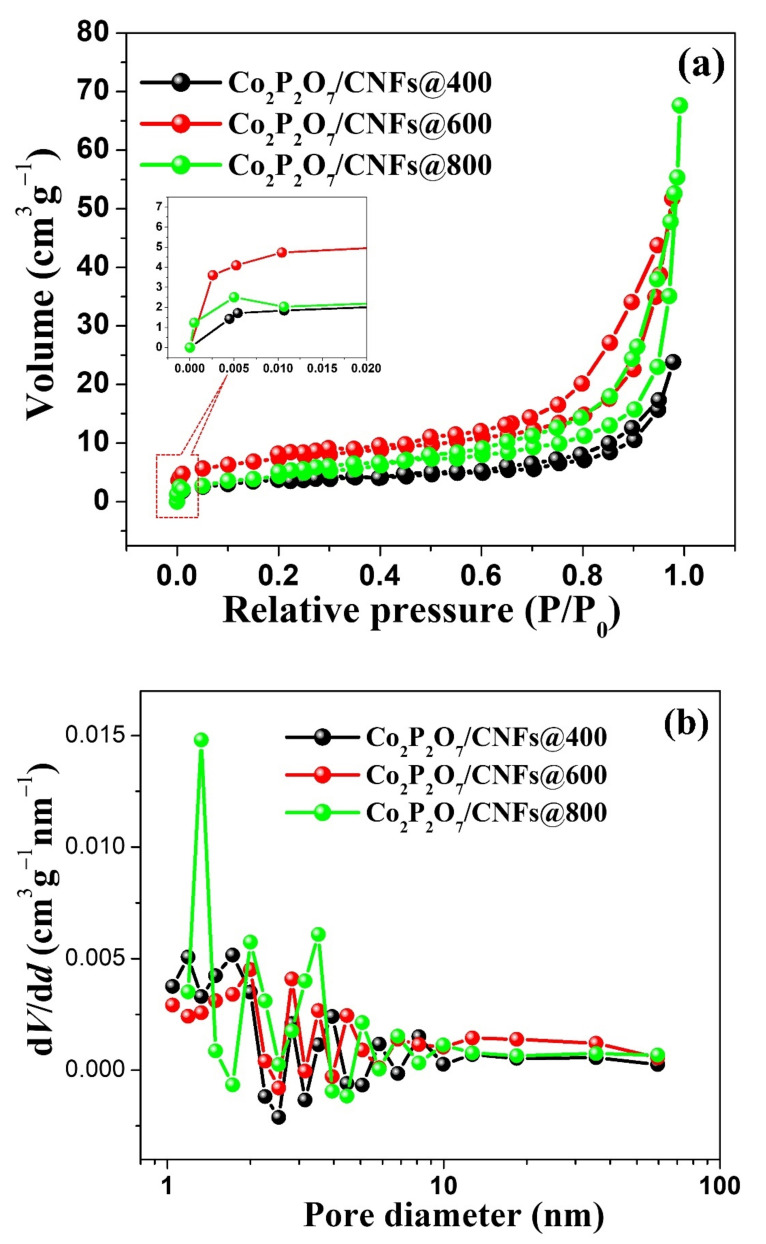
(**a**) N_2_ adsorption–desorption isotherm curves, (**b**) pore–size distribution of Co_2_P_2_O_7_/CNFs@400, Co_2_P_2_O_7_/CNFs@600 and Co_2_P_2_O_7_/CNFs@800.

Many factors affect the performance of supercapacitors, including morphology or shape, porous structure, electrical properties, surface area, surface chemistry, and electrolyte ion sizes. Among these factors, specific surface area (SSA) and pore size distribution (PSD) pay important roles in electrochemical efficiency, as surface area and porosity may increase the number of ions and electron adsorption–desorption, providing enhanced channels for electrolyte diffusion. Nitrogen adsorption–desorption isotherms were examined for all samples to determine their SSA and porosity. The results are shown in Figure 8a. The samples exhibit Type IV adsorption isotherms with H3 hysteresis loops [48]. All samples had microporous and mesoporous features. From the inset of Figure 8a, the adsorption of N_2_ at a low relative pressure (*P/P*_0_ < 0.01) represents the presence of micropores and a consequently higher specific surface area in Co_2_P_2_O_7_/CNFs@600 than in other samples. Then, increased adsorption at higher pressures is indexed to specific surface area features. The specific surface area of all samples was determined at relative pressures of 0.05 < *P/P*_0_ < 0.35 using a Brunauer–Emmett–Teller (BET) method. The SSA values were found to be approximately 13.35, 25.79, and 17.51 m^2^g^−1^ for Co_2_P_2_O_7_/CNFs@400, Co_2_P_2_O_7_/CNFs@600 and Co_2_P_2_O_7_/CNFs@800, respectively. Then, the mesoporous surface area was determined at relative pressures of 0.5 ≤ *P/P*_0_ ≤ 1.0, indicating that Co_2_P_2_O_7_/CNFs@600 have a higher specific surface area due to mesopores than other samples [23,24,26]. Generally, the pore sizes of materials are divided into three groups: macropores (>50 nm), mesopores (2–50 nm) and micropores (<2 nm) [48,49]. Additionally, the mesoporous distribution was examined using a Barrett–Joyner–Halenda (BJH) method from the desorption branch of the isotherm. The results are shown in Figure 8b. All the samples have broad pore diameter distributions, in the range of 2–60 nm. The specific surface areas, pore volumes, and average pore diameters are summarized in Table 2.

### 3.7. Electrochemical Properties

**Figure 9:**
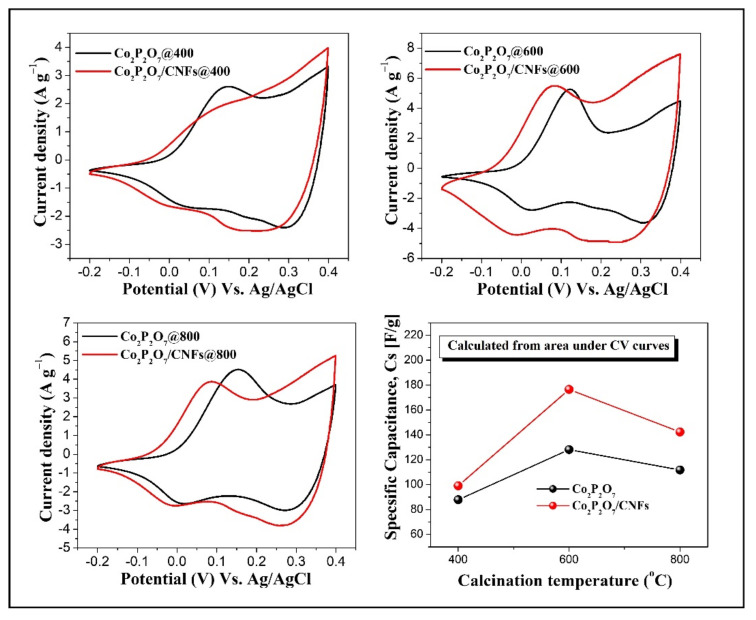
Comparative CV curves at a scan rate of 20 mV s^−1^ with calculated specific capacitance of pure Co_2_P_2_O_7_ versus Co_2_P_2_O_7_/CNFs calcined at 400 °C, 600 °C and 800 °C under an argon atmosphere.

Qualitative information of the relevant electrochemical processes was determined in a 3M KOH electrolyte using cyclic voltammetry (CV). Figure 9 shows comparative CV curves at a scan rate of 20 mV s^−1^ for pure Co_2_P_2_O_7_ versus Co_2_P_2_O_7_/CNFs calcined at 400 °C, 600 °C, and 800 °C under an argon atmosphere. From the CV curves, it can be clearly seen that the shapes of the curves show electrical double layer capacitance (EDLCs) and pseudo–capacitance (PC), indicating that the capacitance mainly results from EDLCs and PC with oxidation and reduction peaks. However, the Co_2_P_2_O_7_/CNFs@400 sample shows a low intensity anodic peak due to its higher EDLCs activity than PC, according to charge accumulation on samples with larger CNF levels [50]. These results are in agreement with the high CNF contents (15.45%) of Co_2_P_2_O_7_/CNFs in the TGA results. The PC capacitance phenomenon of Co_2_P_2_O_7_ is mainly due to a Faradaic effect, which is explained by redox reactions of Co^2+^/Co^3+^ [25,26,51]. Consequently, the redox reaction of Co_2_P_2_O_7_ in KOH electrolyte can be written as Equation (8) [25,26]. Moreover, it can be seen that the CV shape for the Co_2_P_2_O_7_/CNFs@600 electrode is nearly rectangular, indicating that the sample electrode exhibits large storage charge or ions in pore sites. This could be attributed to the porous structure of the Co_2_P_2_O_7_/CNFs@600 electrode. The measured porosity of this sample is in good agreement with the nitrogen adsorption–desorption measurements. Additionally, the areas under the CV curves of all Co_2_P_2_O_7_/CNFs composite electrodes are larger than for pure Co_2_P_2_O_7_ electrodes, demonstrating that the presence of CNF composites played an important role in enhancing electron transfer of electrodes, resulting in higher redox reaction rates [52]. Furthermore, C_s_ values can be calculated using Equation (1) from the area under the CV curves and the obtained results shown in the bottom right of Figure 9. It was found that all the Co_2_P_2_O_7_ composite CNF electrodes have higher C_s_ values than the pure Co_2_P_2_O_7_ electrodes. Additionally, the highest calculated specific capacitance from CV measurements was found in a Co_2_P_2_O_7_/CNFs@600 electrode, 176.5 F g^−1^. Remarkably, the observed anodic peak positions were shifted to lower voltages with composite CNFs electrodes, indicating faster redox reactions on electrode surfaces. For example, a low applied voltage can easily generate current due to the electrons from Co_2_P_2_O_7_ using conductive pathways formed by CNFs. This reduces the charge transfer resistance of the electrode and facilitates movement of ions and electrons [6,27,28,29,30].

Co_2_P_2_O_7_ + 2OH^− ^ ⇆ Co_2_(OH)_2_P_2_O_7_ + 2e^−^ (8)

**Figure 10:**
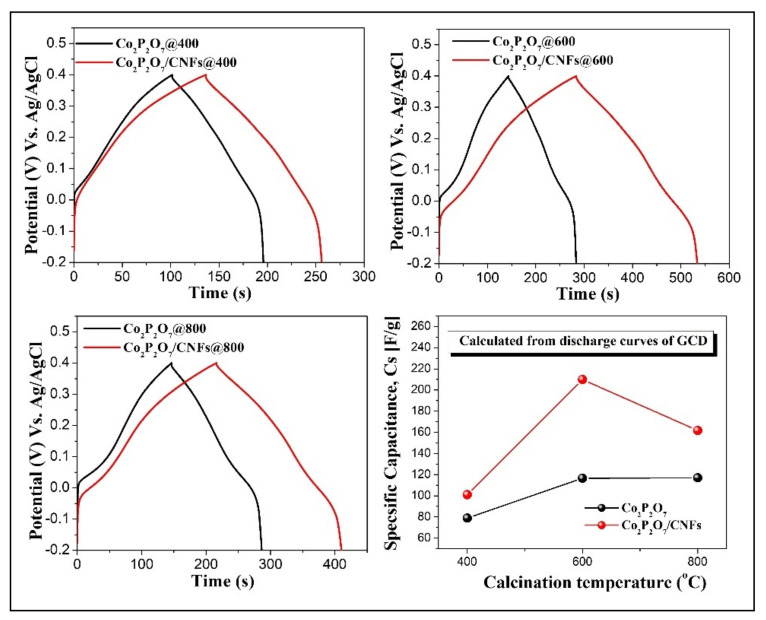
Comparative GCD curves at a current density of 0.5 A g^−1^ with calculated specific capacitances of pure Co_2_P_2_O_7_ versus Co_2_P_2_O_7_/CNFs calcined at 400 °C, 600 °C and 800 °C under an argon atmosphere.

Additionally, the galvanostatic charge–discharge (GCD) at a current density of 0.5 A g^−1^ of pure Co_2_P_2_O_7_ versus Co_2_P_2_O_7_/CNFs calcined at 400 °C, 600 °C, and 800 °C under an argon atmosphere are shown in Figure 10. Symmetric charge–discharge curves combined with EDLCs and PC types were observed. This revealed two energy storage mechanisms, using both ion adsorption as well as fast surface redox reactions [6,11,23]. Furthermore, the C_s_ of each electrode examined using the GCD method was calculated from its discharge curve using Equation (2). These results are shown in the bottom right of Figure 10. It is clear that the Co_2_P_2_O_7_/CNFs@600 electrode exhibited a maximal C_s_, 209.9 F g^−1^. This maximal C_s_ value for Co_2_P_2_O_7_/CNFs@600 can be attributed to the large surface area and high porosity which results in enhanced ion and electron adsorption–desorption. These results are in good agreement with nitrogen adsorption–desorption measurements. Clearly, all the Co_2_P_2_O_7_ composite CNF electrodes have higher C_s_ values than the pure Co_2_P_2_O_7_ electrodes. This suggests that CNF composites can be useful for improving electrochemical performance by increasing the electroactive surface area, enhancing electron transfer, and increasing absorption between the electrode and electrolyte [53,54].

**Figure 11:**
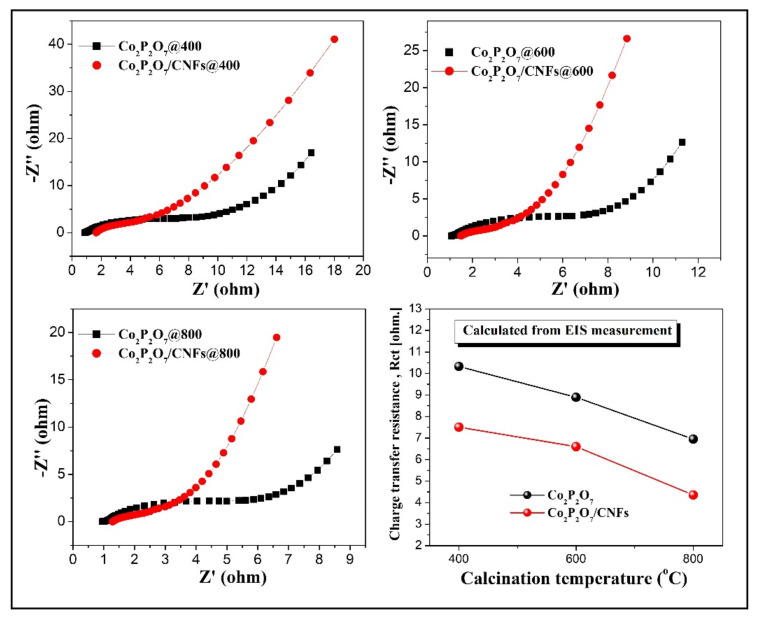
Comparative Nyquist plots with charge transfer resistance (R_ct_) of pure Co_2_P_2_O_7_ versus Co_2_P_2_O_7_/CNFs calcined at 400 °C, 600 °C and 800 °C under an argon atmosphere.

In a further experiment, electrochemical impedance spectroscopy (EIS) measurements were performed to confirm the influence on the conductivity of Co_2_P_2_O_7_/CNFs composites. Nyquist plots of Co_2_P_2_O_7_ versus Co_2_P_2_O_7_/CNFs electrodes are presented in Figure 11. They reveal two overlapping semicircular arcs in the high frequency region and a straight line at low frequencies. Normally, the charge transfer resistance (R_ct_) of an electrode is estimated from the diameter of a semicircle in the high–frequency region of this plot. They were found to be 10.33 Ω (Co_2_P_2_O_7_@400), 8.89 Ω (Co_2_P_2_O_7_@600), and 6.95 Ω (Co_2_P_2_O_7_@600). Then, R_ct_ decreased to 7.51 Ω (Co_2_P_2_O_7_/CNFs@400), 6.60 Ω (Co_2_P_2_O_7_/CNFs@600) and 4.36 Ω (Co_2_P_2_O_7_/CNFs@600) for the CNF composite electrodes. The estimated R_ct_ values of all electrodes are shown at the bottom right of Figure 11. These results indicate that the Co_2_P_2_O_7_ composite CNF electrodes exhibit lower R_ct_ values than the pure Co_2_P_2_O_7_ electrodes. It is notable that the strong bonding in [P_2_O_7_]^4–^ results in a higher energy phosphate bond, also called phosphoric anhydride bonds. This produces strong bonds and good electrical conductivity, resulting in higher conductivity electrodes [55,56,57]. The R_ct_ results are in good agreement with the phase composition in XRD results for samples calcined at 600 °C and 800 °C, indicating that a monoclinic phase with a pyrophosphate group results in strong bonding [P_2_O_7_]^4–^ in these samples. However, an amorphous phase in samples calcined at 400 °C produced higher R_ct_ values than for samples calcined at 600 °C and 800 °C. Additionally, the slope of the line in the lower frequencies region, known as the Warburg resistance (W), presents the diffusion behavior of ions in the electrode pores [58]. Clearly, the slopes of Co_2_P_2_O_7_/CNF composite electrodes are larger than that of the Co_2_P_2_O_7_ electrode, suggesting higher ionic diffusion between the electrode and electrolyte. This EIS analysis demonstrates that the presence of the Co_2_P_2_O_7_/CNF composites played an important role in reducing the charge transfer resistance, resulting in excellent electron transfer by the electrodes.

**Figure 12:**
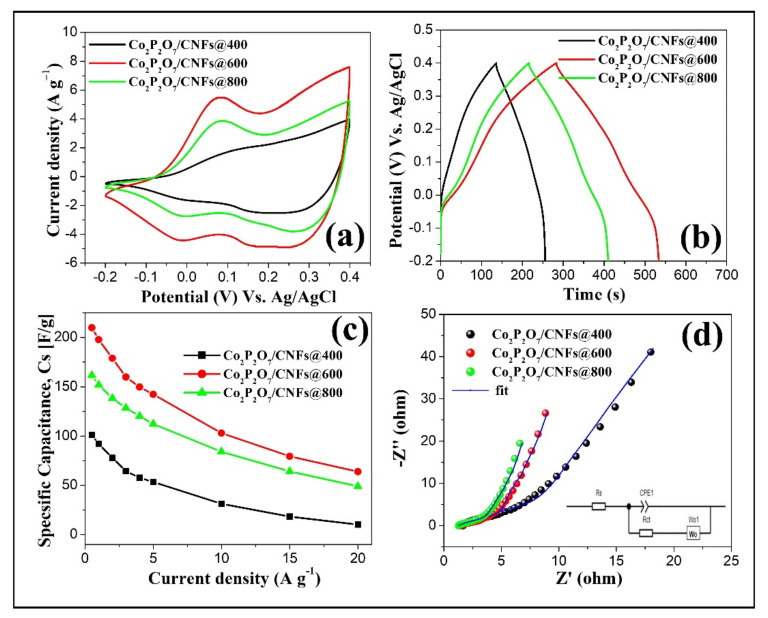
(**a**) CV curves at a scan rate of 20 mV s^−1^, (**b**) GCD curves at a current density of 0.5 A g^−1^, (**c**) specific area capacitance (C_sa_) at various current densities, and (**d**) Nyquist plots for Co_2_P_2_O_7_/CNFs@400, Co_2_P_2_O_7_/CNFs@600, and Co_2_P_2_O_7_/CNFs@800.

In CNF composite electrodes, a comparison of the CV results at a scan rate of 20 mV s^−1^, GCD curves at a current density of 0.5 A g^−1^, the specific area capacitance (C_sa_) at different current densities, and Nyquist plots of Co_2_P_2_O_7_/CNFs@400, Co_2_P_2_O_7_/CNFs@600, and Co_2_P_2_O_7_/CNFs@800 was carried out. As seen in these CV curves (Figure 12a), the redox peaks and areas under the CV curve of the Co_2_P_2_O_7_/CNFs@600 electrode are higher than for other electrodes, indicating that this electrode has a higher redox mechanism and larger C_s_ than the Co_2_P_2_O_7_/CNFs@400 and Co_2_P_2_O_7_/CNFs@800 electrodes. However, a reduction peak was not detected in Co_2_P_2_O_7_/CNFs@400 due to the large charge transfer resistance in an amorphous phase. These results are in good agreement with XRD and EIS measurements, which displayed higher EDLC characteristics than pseudo–capacitance in the CV shape. Additionally, the C_s_ values can be calculated using Equation (1) using the area under the CV curves. They were found to be 99.09, 176.52, and 142.34 F g^−1^ for Co_2_P_2_O_7_/CNFs@400, Co_2_P_2_O_7_/CNFs@600, and Co_2_P_2_O_7_/CNFs@800, respectively. Subsequently, the charge and discharge processes were observed using the GCD method and are shown in Figure 12b. These results indicate that the charge–discharge curve shape for all electrodes was nearly that of EDLCs [34,35]. Additionally, the C_s_ values at various current densities of all electrodes were calculated from the discharge curves using Equation (2). The obtained results are shown in Figure 12c. It was found that the C_s_ values decreased with increased current density. This can be attributed to a higher degree of insufficient Faradic redox reactions and potential drop at higher current densities [16,17,18,19]. Moreover, the Nyquist plots of the Co_2_P_2_O_7_/CNF composites revealed two overlapped semicircular arcs in a high frequency region and a straight line at low frequencies. Additionally, fitting the impedance curves with a constant phase element (CPE), equivalence resistance (R_s_), charge transfer resistance (R_ct_), and ion diffusion resistance (w) using the equivalent circuit is shown in the inset of Figure 12d. From these results, the charge transfer resistances of these electrodes were found to be 7.51, 6.60, and 4.36 Ω for Co_2_P_2_O_7_/CNFs@400, Co_2_P_2_O_7_/CNFs@600, and Co_2_P_2_O_7_/CNFs@800, respectively. The charge transfer resistance of these materials decreased with increasing calcination temperature since CNFs have higher crystallinity and Co_2_P_2_O_7_ has stronger phosphate bonding in [P_2_O_7_]^4^. Clearly, the CNF composite Co_2_P_2_O_7_ electrodes can enhance electron and ion transfer between the surfaces of nanocrystalline Co_2_P_2_O_7_ and an electrolyte resulting in increased energy and power densities.

**Figure 13:**
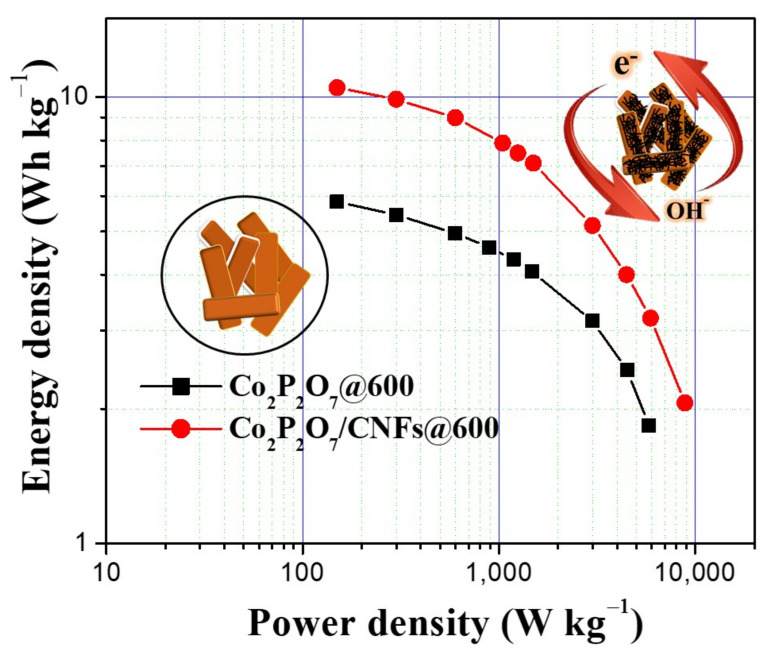
Ragone plot of energy density and power density of Co_2_P_2_O_7_/CNFs@600 compared to Co_2_P_2_O_7_@600.

It is interesting to note that the SSA of the Co_2_P_2_O_7_/CNFs@600 sample (25.8 m^2^g^−1^) is still much lower than the SSA of the pure CNF reported in literature. For instance, Chen et al. reported the SSA of the pyrolyzed–BC CNF as 489.2 m^2^g^−1^ [59]. However, their pure CNF exhibited relatively low capacitance (77.8 F g^−1^) due to the lack of redox reaction. On the other hand, the Co_2_P_2_O_7_ microflowers with the SSA of 13.6 m^2^g^−1^ showed the capacitance of 70 F g^−1^ [26]. Both the SSA and the capacitance are lower than for the Co_2_P_2_O_7_/CNFs@600 in the present work. Additionally, the SSA of the Co_2_P_2_O_7_ nano/microstructures was enhanced to 25.1 m^2^g^−1^ [24]. The SSA value is closed to our work, and thus resulting in the similar capacitance (237.1 F g^−1^). Therefore, it is important to control the pore size and surface area of the electrode samples for maximizing capacitance. Additionally, the excellent electrochemical properties of the Co_2_P_2_O_7_/CNFs@600 nanocomposite electrode can be attributed to two mechanisms. First, high surface area from greater porosity is due to the nature of Co_2_P_2_O_7_. CNFs can increase electron transfer, resulting in faster surface redox reactions [24,34]. Second, the optimal conductivity controlled by adjusting the calcination temperature indicates that the lowest charge transfer resistance, 4.36 Ω, was found in the Co_2_P_2_O_7_/CNFs@800 electrode. However, the C_s_ value of redox materials also depends on surface area and pore structure rather than just conductivity. This combination produced the highest C_s_ in the Co_2_P_2_O_7_/CNFs@600 electrode [6,22]. It is clear from this work that the improved electrochemical properties of the Co_2_P_2_O_7_/CNFs@600 nanocomposite electrode can be attributed to the excellent pyrophosphate properties of Co_2_P_2_O_7_ and good electron transfer via CNF composite electrodes. Likewise, the stored charge mechanisms of the Co_2_P_2_O_7_/CNF electrodes are achieved greatly by ion adsorption (EDLCs) and fast surface redox reactions (pseudo–capacitance) [6,11,23]. Therefore, the relative contribution from EDLCs and pseudo–capacitance can be adjusted by calcination temperature, which allows the ability to customize the properties of their energy storage. In the case of the Co_2_P_2_O_7_/CNF@600 electrode, it exhibits higher specific micro– and mesoporous surface areas and consequently a higher specific surface area than other electrodes. The largest specific surface area and total pore volumes of the Co_2_P_2_O_7_/CNF@600 electrode can improve the electro–active sites of the electrode [24,34,60]. This results in the enhanced redox reaction and ion adsorption, which show the highest specific capacitance of 209.9 F g^−1^ at a current density of 0.5 A g^−1^. Additionally, a Ragone plot for Co_2_P_2_O_7_/CNFs@600 compared to Co_2_P_2_O_7_@600 for their corresponding energy and power densities as supercapacitors presents a high power density, 8850 W kg^−1^, with a maximum energy density of 10.5 Wh kg^−1^, as shown in Figure 13.

## 4. Conclusions

In summary, Co_2_P_2_O_7_/CNFs composites were successfully prepared via calcination after a hydrothermal process employing NH_4_CoPO_4_∙H_2_O/BC. The crystal structure, thermal analysis, morphology, and pore structure of Co_2_P_2_O_7_/CNFs were investigated by various methods, such as XRD, FE–SEM, TGA–DTG, TEM, FT–IR, and N_2_ adsorption–desorption. As a result, the largest specific surface area and pore structure were observed for the Co_2_P_2_O_7_/CNF@600 electrode due to the improved electro–active sites in the electrode, resulting in the enhanced redox reaction and ion adsorption. The Co_2_P_2_O_7_ composite with CNFs can reduce the charge transfer resistance of electrodes through electron transfer optimization, which indicates an improvement in electron transport capacity. Additionally, by using an optimal calcination temperature, the Co_2_P_2_O_7_/CNFs@600 material exhibits a maximal specific capacitance. Furthermore, high electrical conductivity can decrease the charge transfer resistance of the electrodes, thus leading to an increased power density. The results of using nanocrystalline Co_2_P_2_O_7_ and carbon nanofiber (Co_2_P_2_O_7_/CNFs) composites suggests that CNFs provide electrical conductivity created during the redox reactions of Co_2_P_2_O_7_.

## Figures and Tables

**Table 1:** Atomic weight (%) of C, O, P and Co in Co_2_P_2_O_7_/CNFs@400, Co_2_P_2_O_7_/CNFs@600 and Co_2_P_2_O_7_/CNFs@800.

Element	Atomic Weight (%)		
	Co_2_P_2_O_7_@400	Co_2_P_2_O_7_@600	Co_2_P_2_O_7_@800
C (K)	18.5 ± 0.3%	16.9 ± 0.4%	7.3 ± 0.3%
O (K)	54.3 ± 0.4%	53.8 ± 0.5%	57.2 ± 0.5%
P (K)	13.9 ± 0.2%	15.9 ± 0.3%	16.8 ± 0.3%
Co (K)	13.3 ± 0.3%	13.5 ± 0.4%	18.7 ± 0.5%

**Table 2:** Summary of specific surface area, pore volume and average pore diameters of Co_2_P_2_O_7_/CNFs@400, Co_2_P_2_O_7_/CNFs@600 and Co_2_P_2_O_7_/CNFs@800.

Samples	BET Specific Surface Area (m^2^g^−1^)	Mesopore Analysis			Micropore Analysis		
		Pore Specific Surface Area (m^2^g^−1^)	Pore Volume (cm^3^ g^−1^)	Average Pore Diameter (nm)	Pore Specific Surface Area (m^2^g^−1^)	Pore Volume (cm^3^ g^−1^)	Average Pore Diameter (nm)
Co_2_P_2_O_7_/CNFs@400	13.4	18.8	0.0335	7.55	10.8	0.0227	1.9
Co_2_P_2_O_7_/CNFs@600	25.8	25.8	0.0734	11.4	27.2	0.0660	1.5
Co_2_P_2_O_7_/CNFs@800	17.5	21.4	0.0979	18.4	11.9	0.0295	1.3

## Data Availability

The data presented in this study are available on request from the corresponding author.

## Supplementary Materials

The following are available online at https://www.mdpi.com/article/10.3390/nano11082015/s1, Figure S1: XRD patterns of (a) Co2P2O7@400, Co2P2O7@600 and Co2P2O7@800 and (b) BC, CNFs@400, CNFs@600 and CNFs@800 with compared to the references for cellulose, Figure S2: FE–SEM images with histograms of diagonal size distribution of (a) Co2P2O7/CNFs@400, (b) Co2P2O7/CNFs@600 and (c) Co2P2O7/CNFs@800, Table S1: Values of fitting parameters obtained from the EIS analysis.
