# Supplementary material for: Co2P2O7 Microplate/Bacterial Cellulose–Derived Carbon Nanofiber Composites with Enhanced Electrochemical Performance
Source: Nanomaterials (Basel). 2021 Aug 6;11(8):2015. doi: 10.3390/nano11082015 (PMC8400182; doi:10.3390/nano11082015)
Supplement: Supplementary file 1 [file nanomaterials-11-02015-s001.zip › nanomaterials-1317871-supplementary.pdf]

Supplementary Information

$Co_2P_2O_7$ Microplate/Bacterial Cellulose–Derived Carbon Nanofiber Composites with Enhanced Electrochemical Performance

Likkhasit Wannasen [1], Wiyada Mongkolthanaruk [2], Ekaphan Swatsitang [1], Prasert Pavasant [3] and Supree Pinitsoontorn [1,*]

[1] Institute of Nanomaterials Research and Innovation for Energy (IN–RIE), Department of Physics, Faculty of Science, Khon Kaen University, Khon Kaen 40002, Thailand; likkhasit123@hotmail.co.th (L.W.); ekaphan@kku.ac.th (E.S.)

[2] Department of Microbiology, Faculty of Science, Khon Kaen University, Khon Kaen 40002, Thailand; wiymon@kku.ac.th

[3] Thai Roong Ruang Sugar Group, Bangkok 10120, Thailand; supersert@gmail.com

* Correspondence: psupree@kku.ac.th; Tel.: +66–43–203-166; Fax: +66–43–202-374

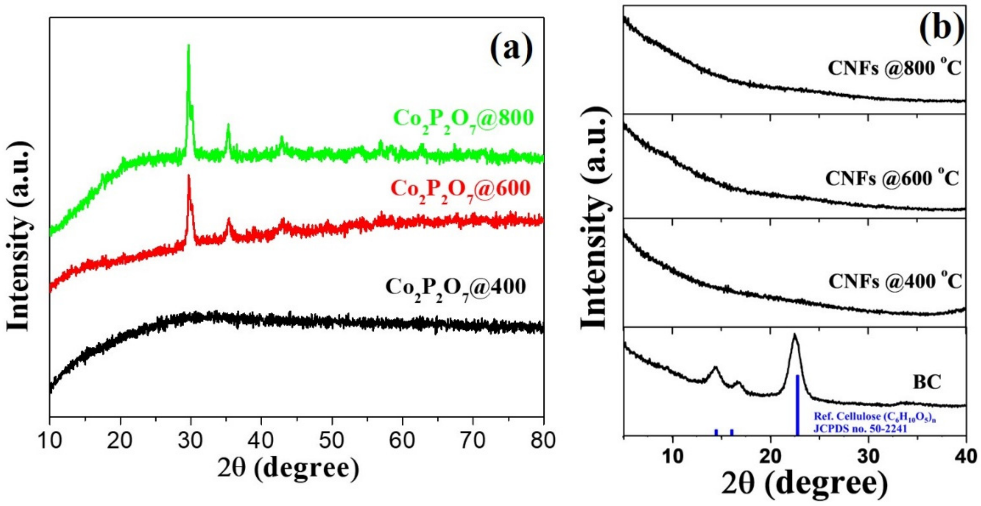

Figure S1 XRD patterns of **(a)** $Co_2P_2O_7$@400, $Co_2P_2O_7$@600 and $Co_2P_2O_7$@800 and **(b)** BC, CNFs@400, CNFs@600 and CNFs@800 with compared to the references for cellulose.

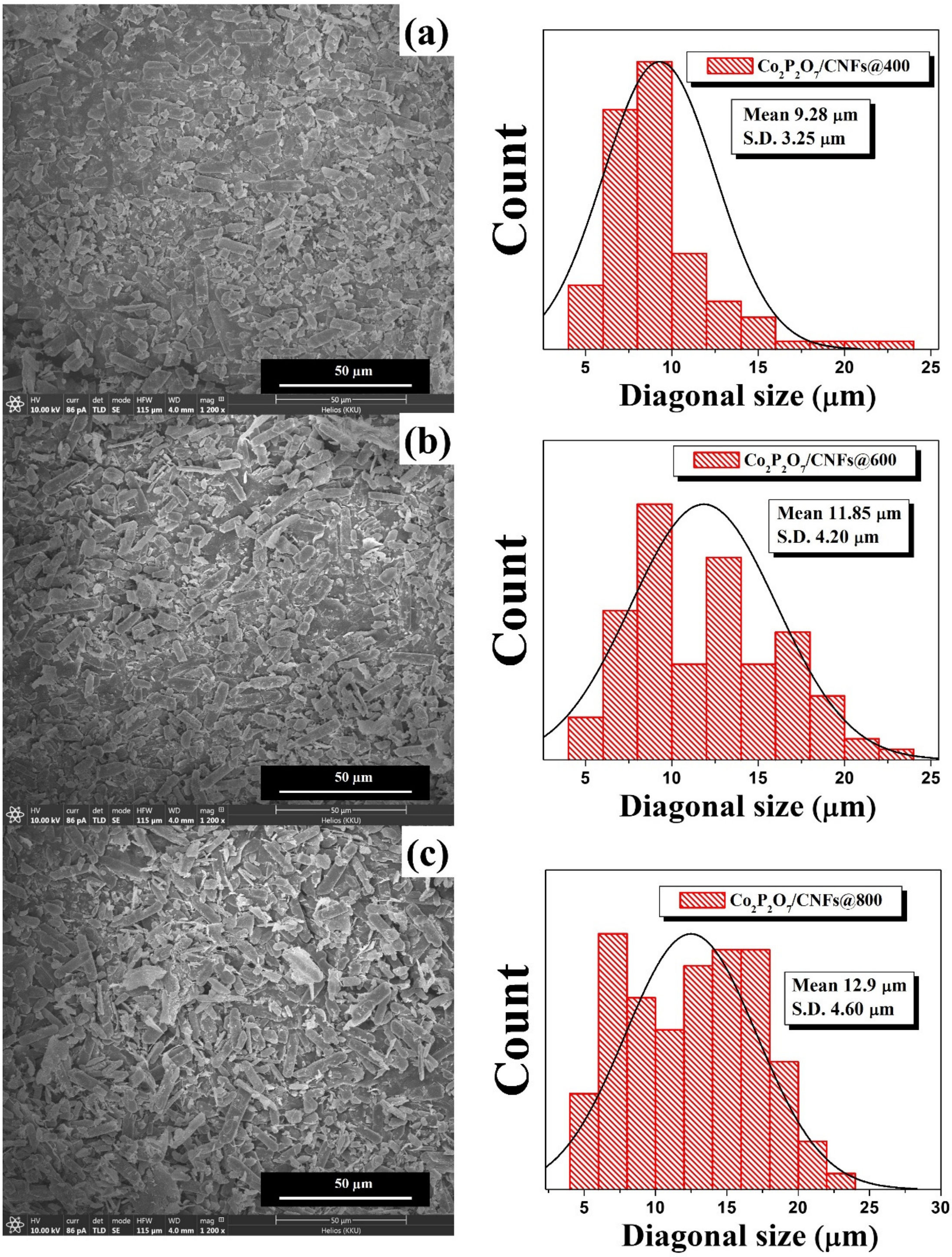

Figure S2 FE-SEM images with histograms of diagonal size distribution of **(a)** $Co_2P_2O_7$/CNFs@400, **(b)** $Co_2P_2O_7$/CNFs@600 and **(c)** $Co_2P_2O_7$/CNFs@800.

Table S1 Values of fitting parameters obtained from the EIS analysis.

Parameters	$Co_2P_2O_7$/CNFs@400	$Co_2P_2O_7$/CNFs@600	$Co_2P_2O_7$/CNFs@800
**R_s (Ω)**	1.6378±0.67%	1.4536± 0.69%	1.274±0.56%
**CPE1-T**	0.064873±2.38%	0.16174±1.28%	0.18619±0.90%
**CPE1-P**	0.67669±0.85%	0.52697±0.85%	0.61347±0.53%
**R_{ct} (Ω)**	7.5071±3.96%	6.6023±4.27%	4.3573±1.98%
**Wo1-R**	15.174±1.83%	14.184±2.38%	13.986±1.60%
**Wo1-T**	2.7811±4.10%	2.6875±6.75%	2.5714±1.41%
**Wo1-P**	0.45138±1.20%	0.55703±1.23%	0.56201±0.67%
